# Unraveling role of arginine-NO metabolism by targeting HIF-1α signaling in mediating esophageal squamous cell carcinoma

**DOI:** 10.3389/fmolb.2026.1873933

**Published:** 2026-07-13

**Authors:** Kaiyuan Yao, Shunshun Zhang, Siya Tang, Yalin Zhang, Xuewei Zheng, Qinan Yin, Chengcheng Tao, Anshun Zhao

**Affiliations:** 1 Key Laboratory of Microbiome and Esophageal Cancer Prevention and Treatment, The First Affiliated Hospital, and College of Clinical Medicine of Henan University of Science and Technology, Luoyang, China; 2 Institute of Organoid on Chip and Drug Translation Research, Henan Academy of Sciences, Zhengzhou, China; 3 Precision Medicine Laboratory, School of Medical Technology and Engineering, Henan University of Science and Technology, Luoyang, China; 4 College of Pharmacy, Chongqing Medical University, Chongqing, China

**Keywords:** arginine metabolism, esophageal cancer, HIF-1α, iNOS, metabolomics, nitric oxide

## Abstract

**Objective:**

Due to the lack of specific biomarkers, patients with esophageal cancer are often diagnosed at an advanced stage, resulting in poor treatment outcomes. This study aims to identify potential diagnostic and therapeutic biomarkers for esophageal squamous cell carcinoma (ESCC) through metabolomics and to elucidate their mechanisms of action.

**Methods:**

Untargeted metabolomics was employed to analyze ESCC tissue samples and matched normal esophageal tissue samples. Differentially expressed metabolites were identified using multivariate statistical analysis. MetaboAnalyst 6.0 software was used for pathway analysis. Key molecules were validated via immunohistochemistry (IHC) and Western blot (WB), and their functions were assessed through cellular functional assays.

**Results:**

A total of 2,850 metabolites were identified, among which 939 were differentially regulated, including 575 upregulated and 364 downregulated metabolites. Pathway enrichment analysis revealed that these differentially expressed metabolites were predominantly enriched in amino acid metabolism-related pathways, Mechanistically, activation of the HIF-1α–iNOS–NO signaling axis drove arginine metabolic reprogramming, leading to profound metabolic disturbances that ultimately enhanced the migratory and invasive capacities of ESCC cells.

**Conclusion:**

This study confirms that ESCC exhibits significant amino acid metabolic dysregulation, with arginine related metabolic pathways being significantly enriched. This disruption in arginine metabolism may be caused by hypoxia, which activates the iNOS-NO signaling axis mediated by HIF-1α, thereby promoting the migratory and invasive capabilities of ESCC. Finally, the study confirmed that NO can serve as a potential non-invasive biomarker for distinguishing ESCC.

## Graphical Abstract

**Figure:**
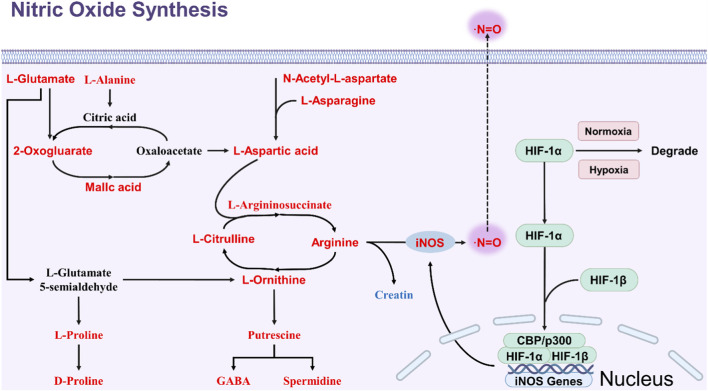
Arginine metabolism mediated by HIF-1α–iNOS–NO signaling enhance ESCC.

## 1 Introduction

Esophageal cancer (EC) ranks seventh among the leading causes of cancer-related deaths worldwide (Bray et al., 2024), with approximately 90% of cases being esophageal squamous cell carcinoma (ESCC) (Smyth et al., 2017). The main clinical challenge is that most ESCC patients receive a late-stage diagnosis, primarily due to the lack of specific biomarkers required for early detection. Currently, gastroscopy serves as the mainstream screening method for ESCC. However, its high-cost limits accessibility for most patients, ultimately contributing to the disease’s high incidence and mortality rates (Lu et al., 2025). Against this backdrop, the emerging technology of metabolomics demonstrates significant potential in identifying specific metabolite biomarkers associated with cancer diagnosis, stratification, prognosis, and treatment (Cai et al., 2025). Unlike genomics and transcriptomics, which primarily predict “potential changes” in biological processes, metabolomics directly reflects “changes that have already occurred” by analyzing endogenous metabolite profiles. This unique advantage makes it particularly well-suited for revealing actionable biomarkers for diseases such as ESCC.

Metabolomics testing has been applied in early screening, diagnosis, and treatment research for tumors in organs such as the oral cavity (Liu Y. et al., 2025; Rajneesh et al., 2025), stomach (Yang et al., 2025), and colon and rectum (Wang et al., 2025; Zheng et al., 2026). Metabolomics has been widely applied in EC. Asamitsu et al. performed sulfur metabolomics analysis on blood samples and exhaled breath condensate (EBC) from EC patients and healthy controls using tandem mass spectrometry, identifying cysteine hydropersulfide (CysSSH) from EBC as a promising non-invasive biological marker (Asamitsu et al., 2025). Sun et al. revealed significant alterations in the purine recycling pathway in ESCC through multi-omics sequencing. They found that hypoxanthine/xanthine abundance positively correlated with tumor size and established a diagnostic model based on these two-purine recycling-related metabolites (Sun et al., 2024). Gao et al. integrated metabolomics and microbiomics technologies to analyze fecal samples from ESCC patients and healthy controls, confirming that phenylethanolamine and N-deoxypropoxybenzene are reliable biomarkers (Gao M. et al., 2024).

In addition to identifying biomarkers for diagnosis, metabolomics can also identify biomarkers to guide treatment. Wu et al. employed GC-MS metabolomics to analyze serum samples from ESCC patients before and after concurrent chemoradiotherapy. By integrating Olink proteomics technology, they constructed a model that effectively predicts prognosis and hematologic toxicity, revealing the key role of L-serine (Wu et al., 2025). Lin et al. established a predictive model based on sphingolipids and triglycerides to assess the therapeutic efficacy in ESCC (Lin et al., 2024). Metabolomics samples can be derived from exhaled breath (Segrado et al., 2025), peripheral blood (Chen et al., 2026), bile (Xia et al., 2025), urine (Carapito et al., 2026), feces (Gao M. et al., 2024), and tissues (Liang et al., 2019), offering flexible options for clinical application.

Hypoxia-inducible factor-1α (HIF-1α) is a key transcriptional regulator involved in cellular adaptation to hypoxic conditions. Under hypoxic conditions, HIF-1α is stabilized and translocated to the nucleus by inhibiting ubiquitin-proteasome degradation mediated by prolyl hydroxylases (PHDs), subsequently forming a heterodimer with HIF-1β to activate the transcription of downstream target genes (Gou et al., 2026). These target genes are extensively involved in angiogenesis (e.g., VEGF) (Zhou et al., 2024), glucose metabolic reprogramming (e.g., GLUT1, LDHA), epithelial-mesenchymal transition (EMT) (Cui et al., 2016), cell proliferation, resistance to apoptosis, and chemoresistance, among other processes. Therefore, HIF-1α is considered a key molecular hub linking the hypoxic tumor microenvironment with malignant biological behavior. In digestive system tumors, HIF-1α is overexpressed in various malignancies, including EC, gastric cancer, liver cancer, and colorectal cancer, and its expression levels are closely associated with tumor proliferation, invasion (Hu et al., 2020; Gao A. et al., 2024), drug resistance, and poor prognosis (Gou et al., 2026).

Arginine is a semi-essential amino acid that participates in numerous key metabolic pathways in living organisms. Abnormal arginine metabolism in tumor cells has become one of the key hallmarks of metabolic reprogramming. In the tumor microenvironment, abnormal arginine metabolism not only affects the proliferation and survival of tumor cells themselves but also reshapes the immune microenvironment by regulating the function of immune cells (such as T cells, macrophages, and myeloid suppressor cells), thereby promoting tumor immune evasion (Bai et al., 2025). Therapeutic strategies based on arginine metabolism, such as arginine deprivation therapy, have demonstrated promising preclinical and clinical efficacy in various tumors (Tomlinson et al., 2015; Tian et al., 2026; Wang et al., 2026).

**FIGURE 1:**
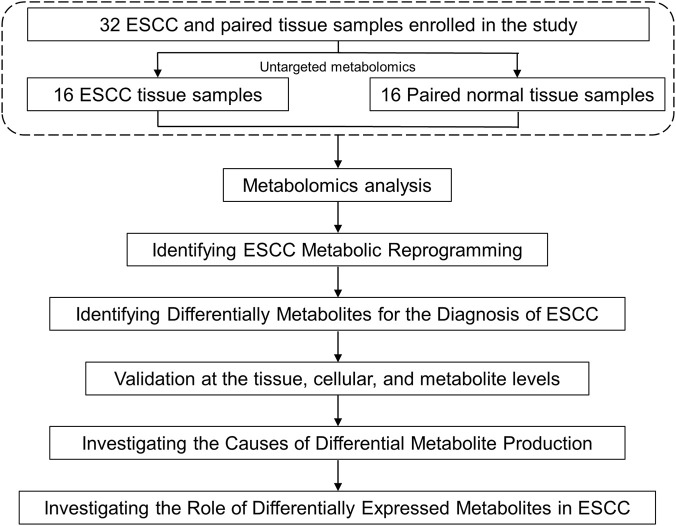
Schematic diagram of this study.

Despite these advances, the unique abnormal metabolic pathways specific to ESCC and their regulatory roles in disease progression remain incompletely elucidated. Here, we adopted an integrated research strategy involving discovery via unsupervised metabolomics, validation via targeted approaches, and functional confirmation. First, we used unsupervised metabolomics to screen for differentially expressed metabolites (DEMs) and pathways between ESCC and normal tissues. We then preliminarily validated these findings using IHC on clinical samples. Finally, we elucidated the function of the HIF-1α-iNOS-NO axis through pharmacological interventions combined with cellular functional assays. This study aims to identify unique aberrant metabolic pathways present in ESCC compared to normal esophageal tissue, explore their functional roles in ESCC progression, and further investigate their potential diagnostic and therapeutic applications in this disease. Figure 1 illustrates the technical roadmap of this paper.

## 2 Materials and methods

### 2.1 Patient source and sample collection

ESCC and paired normal esophageal tissues (≥5 cm from the tumor margin, pathologically confirmed to be free of cancer cell infiltration) were collected for liquid chromatography-tandem mass spectrometry (LC-MS/MS) analysis. All tissue samples were obtained from the First Affiliated Hospital of Henan University of Science and Technology.

Patients were diagnosed with ESCC via preoperative gastroscopy and subsequently enrolled in the study. ESCC tissues were harvested during surgery and confirmed by postoperative pathological biopsy. All patients had not received chemotherapy, radiotherapy, or any other anti-tumor drug treatment prior to tumor resection. Written informed consent was obtained from enrolled patients before participation. This study was conducted in accordance with the Declaration of Helsinki and approved by the Ethics Committee of the First Affiliated Hospital of Henan University of Science and Technology (FAH-HAUST) (Approval No.: 2025-0273). Tissue samples were immediately frozen and stored after collection, followed by sample preparation and extraction as described below.

### 2.2 Sample preparation and extraction

Thaw the tissue samples slowly on ice. Weigh 100 mg of tissue sample slowly and accurately onto ice into a centrifuge tube, then add 200 μL of pre-chilled ultrapure water. After vortexing for 60 s, 0.8 mL of pre-chilled methanol-acetonitrile mixture (1:1, v/v) was added and vortexed again for 60 s. Ultrasonic extraction was performed at low temperature for 30 min. At −20 °C, the protein was allowed to precipitate for 1 h. Subsequently, the sample was centrifuged at 12,000 rpm and 4 °C for 10 min. The supernatant was freeze-dried in a freeze-dryer at −80 °C. The dried residue was vortexed with 200 μL of a 30% acetonitrile-water solution (v/v) and centrifuged at 14,000 rpm at 4 °C for 15 min. Finally, the supernatant was collected for instrumental analysis.

### 2.3 Untargeted metabolomics analysis

The liquid chromatography-mass spectrometry system (LC/MS) was used for untargeted metabolomics analysis. LC parameters are as follows: Column: Waters HSS T3 (100 × 2.1 mm, 1.8 μm). Mobile phase: Phase A (ultrapure water containing 0.1% formic acid, v/v); Phase B (acetonitrile containing 0.1% formic acid, v/v). Injection volume: 2 μL. Flow rate: 0.3 mL/min. Column temperature: 40 °C. Elution gradient: 0 min (100% A, 0% B); 1 min (100% A, 0% B); 1–12 min (linear gradient from 100% A to 5% A, 0% B to 95% B); 12–13 min (5% A, 95% B); 13–13.1 min (rapid return to 100% A, 0% B); 13.1–17 min (100% A, 0% B, column equilibration). All samples were maintained in a 4 °C autosampler during analysis. To avoid systematic errors caused by instrument signal fluctuations, samples were analyzed continuously in random order. Quality control samples were used to monitor and evaluate system stability and the reliability of experimental data.

MS parameters are as follows: A Q Exactive HFX high-resolution mass spectrometer (Thermo, USA) equipped with an electrospray ionization (ESI) source was used for primary and secondary mass spectrometry data collection. The parameters were set as follows: sheath gas pressure = 40 arb; auxiliary gas pressure = 10 arb; spray voltage = +3000 V (positive ion mode)/-2800 V (negative ion mode); capillary temperature = 350 °C; ion transfer tube temperature = 320 °C; scan mode = Full-ms-ddMS2; mass range = m/z 70–1050; primary resolution = 70,000 FWHM; secondary resolution = 17,500 FWHM.

### 2.4 Qualitative and quantitative analysis of metabolites in ESCC and normal tissues

Raw data were preprocessed using Progenesis QI software (Waters Corporation, Milford, USA) for baseline correction, peak detection, peak matching, retention time alignment, and peak alignment, resulting in a data matrix comprising retention times, mass-to-charge (m/z) ratios, and peak intensities. Features associated with MS/MS spectra were annotated using commercial databases, SanShu Bio’s proprietary metabolite MS/MS database, and matching fragmentation patterns. The quality of MS/MS spectral matching was evaluated using a fragmentation score ranging from 0 to 1, with scores greater than 0.5 considered indicative of reliable metabolite annotation.

Using a local metabolite database, the metabolites underwent mass spectrometry-based qualitative and quantitative analysis. The multi-peak MRM metabolite detection spectrum displays detectable substances in the samples, with each differently colored chromatographic peak representing a detected metabolite. Characteristic ions for each compound were identified via triple quadrupole screening. Signal intensity for these characteristic ions was obtained in the detector. Analyze peak integration and calibration using the MultiQuant software. The peak area for each chromatographic peak represents the relative abundance of the corresponding compound.

Multivariate statistical analysis was used to distinguish specific metabolites between ESCC and normal tissues. DEMs were screened with the criteria: Student’s t-test P-value <0.05 and Variable Importance in the Projection (VIP) >1 (from the first principal component of the OPLS-DA model). Pathway enrichment analysis was performed using MetaboAnalyst 6.0 with Kyoto Encyclopedia of Genes and Genomes (KEGG) pathway annotation.

Finally, pathway analysis was performed using MetaboAnalyst 6.0 by integrating KEGG database based on the DEMs.

### 2.5 Immunohistochemistry (IHC)

We collected cancer and non-cancer tissue samples from 11 patients with ESCC treated at FAH-HAUST between 2023 and 2025. The tissues were fixed in paraformaldehyde and embedded in paraffin. Continuous sections were prepared prior to experimentation. The primary antibody iNOS Rabbit pAb (Immunoway, YT3169) was incubated overnight at 4 °C (diluted 1:50 in TE buffer, pH 9.0). DAB staining was conducted using the DAB staining kit (ZSGB-BIO, PV-9000). Results were scored by two pathologists blinded to clinical and pathological data.

### 2.6 Cell culture

ESCC cell lines (KYSE-30, KYSE-150) and NE6-T were obtained from the Tumor Molecular Biology Laboratory of the FAH-HAUST. ESCC cell lines were cultured in RPMI 1640 medium, while NE6-T cells were cultured in high-glucose DMEM medium. All media were supplemented with 10% fetal bovine serum (FBS) and 1% penicillin-streptomycin. All cells were cultured in a humidified incubator at 37 °C with 5% CO_2_. For subsequent experiments, cells were treated with CoCl_2_ (150 μmol/L, HIF-1α inducer) or LW6 (10 μmol/L, HIF-1α specific inhibitor, MCE, Cat. No. 934593-90-5).

### 2.7 Western blot (WB) analysis

Cells in the logarithmic growth phase were harvested and seeded into 10 cm cell culture dishes. They were cultured in a CO_2_ incubator until reaching 80% confluence. CoCl_2_ and LW6 were added, and the experiment was terminated after 24 h of incubation. Total cellular proteins were extracted on ice using a cell lysis buffer (PMSF: RIPA = 1:100). Protein concentrations were determined using the BCA method. Equal amounts of protein (30 μg per lane) were separated by SDS-PAGE. Proteins were separated by SDS-PAGE (upper gel: 90 V for 30 min; lower gel: 120 V for 2 h). Subsequently, the proteins were transferred to a PVDF membrane (250 mA for 1.5 h). The membrane was blocked with 5% skim milk and incubated at 4 °C with anti-iNOS (1:1000, YT3169, Immunoway) and anti-GAPDH (1:20000, 60004-1-Ig, Proteintech) primary antibodies at 4 °C. The following day, after washing with TBST, the PVDF membrane was incubated at room temperature with the corresponding secondary antibody (1:5,000, SA00001-2, Proteintech). Finally, protein bands were visualized using a highly sensitive ECL chemiluminescence kit. Band intensities were quantified using ImageJ software. All experiments were independently repeated three times, and representative blots are shown.

### 2.8 NO content determination

Cells were seeded at a density of 5 × 10^6^ cells/well in 10 cm dishes and cultured in a cell culture incubator until reaching 80% confluence. Subsequently, CoCl_2_, LW6, and complete medium (as the control group) were added separately. The experiment was terminated after 24 h of incubation in the incubator. Cells were lysed using Cell and Tissue Lysis Buffer (Beyotime, S3090). NO levels in the cells were detected using the NO Detection Kit (Beyotime, S0021S). Determine the NO concentration in the sample based on the standard curve.

### 2.9 Wound healing assay

Seed cells at a density of 5 × 10^5^ cells per well into a six-well cell culture plate. Incubate at 37 °C in a 5% CO_2_ incubator until cells reach 90% confluence, forming a uniform monolayer. Using the tip of a sterile 200 μL pipette, streak each well by drawing vertical lines perpendicular to the bottom of the 6-well plate. Gently wash four times with PBS to remove detached cells. Add serum-free medium, followed by CoCl_2_ or LW6. Incubate for 24 h before terminating the experiment. Capture images at 0, 6, 12, and 24 h using an inverted microscope. Measure migration area using ImageJ software and calculate cell migration rate with GraphPad Prism 9.0.

### 2.10 Transwell assay

Starve well-grown cells in RPMI 1640 medium for 1 day. Thaw the matrix gel overnight at 4 °C. Pre-cool pipettes, tips, and PBS at 4 °C. Dilute the matrix gel with pre-cooled serum-free 1640 medium to 1 mg/mL.

Using chilled pipette tips, dispense 100 μL of matrix gel (1 mg/mL) into each Transwell chamber, spreading it evenly across the bottom without creating bubbles. Add 100 μL RPMI 1640 medium to each Transwell chamber and incubate at 37 °C for 30 min to hydrate the membrane. Remove the liquid from the chambers and check for fluid migration through the membrane. If none is observed, proceed with cell seeding. Add 500 μL of complete medium to the lower chamber of the 24-well plate.

Digest the starved cells, resuspend in RPMI 1640 medium, and gently blow and mix thoroughly with a pipette to avoid cell aggregation. Count cells and adjust cell density to 2 × 10^5^ cells/mL. Add 100 μL of cell suspension to each upper chamber. Place the 24-well plate in a cell culture incubator. After 1 day, remove only the Transwell chambers. Remove the top layer of cells. Add 4% paraformaldehyde fixative to clean wells in a 24-well plate, then place the Transwell chambers into the wells containing the fixative to preserve cell morphology. After 30 min, wash the Transwell chamber three times with pre-chilled PBS, for 5 min each time. Next, add 0.1% crystal violet staining solution to clean wells of a 24-well plate, place the Transwell chamber into the well containing the staining solution, and stain for 10 min. Wash the Transwell chamber three times with pre-chilled PBS, for 5 min each time, allow to air-dry briefly, then observe and photograph under a microscope.

### 2.11 Statistical analysis

Data are presented as mean ± SEM. A paired t-test was used for paired tissues. One-way ANOVA with Tukey’s post-hoc test was used for multiple comparisons. P < 0.05 was considered significant.

## 3 Results

### 3.1 Patient and clinical characteristics

Between January 2023 and October 2025, clinical samples meeting the following criteria were collected from FAH-HAUST: postoperative pathological diagnosis of ESCC; no prior preoperative chemotherapy, radiotherapy, or other anti-tumor treatments; and complete clinical and pathological data. This study included a total of 32 patients with ESCC, with cancerous tissue and matched adjacent normal tissue (≥5 cm from the tumor margin, pathologically confirmed to be free of cancer cell infiltration) collected. All samples underwent metabolomic profiling using LC-MS/MS technology.

### 3.2 The amino acid metabolism disorder in ESCC patients

Metabolites in normal and tumor tissues were identified using LC-MS/MS, and a total of 2,850 metabolites were successfully identified.

**FIGURE 2:**
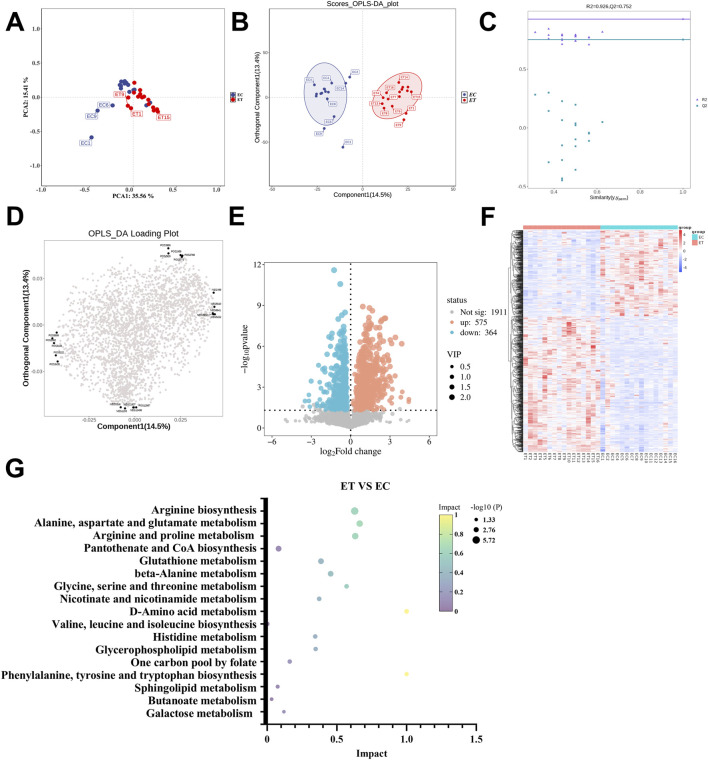
Statistical Analysis and Pathway Analysis in Untargeted Metabolomics. **(A)** PCA plot; **(B)** OPLS-DA score plot; **(C)** OPLS-DA permutation test plot; **(D)** OPLS-DA loadings plot; **(E)** Volcano plot; **(F)** Heatmap of differentially expressed metabolites; **(G)** Pathway analysis plots.

PCA scatter plot indicates the holistic distribution of ESCC (red, ET) and adjacent normal tissue (blue, EC) (Figure 2A). The OPLS-DA scatter plot further verifies the overall distribution of the tissue samples. The OPLS-DA shuffle plot displays the model parameters (R2 = 0.926, Q2 = 0.752), demonstrating the model’s reliability, absence of overfitting, and high predictive power (Figures 2B,C). Figure 2D lists the top 20 metabolites with the highest contribution scores. Both results indicate the specific metabolic patterns between normal and tumor tissues.

Combined with the results from the volcano plot, we identified 939 significantly differentially expressed metabolites between ESCC tissue and normal tissue. Compared to matched normal esophageal tissue, 575 metabolites were upregulated, and 364 were downregulated in ESCC tissue (Figure 2E). Figure 2F shows the relative abundance of metabolites across 32 samples. This heatmap reveals a clear separation between ESCC and normal tissues based on the metabolic profiles, with each group exhibiting distinct clustering patterns. To further identify the potential function of the DEMs, the pathway analysis was performed. A total of 17 metabolic pathways were significantly enriched (Figure 2G). Among them, multiple amino acid-related pathways were significantly enriched between both groups, including arginine biosynthesis; alanine, aspartic acid, and glutamic acid metabolism; and arginine and proline metabolism (Figure 2G). The results suggested that patients with ESCC exhibit significant amino acid metabolic disorders, hinting that amino acid metabolic disorders may underlie the initiation and progression of ESCC.

### 3.3 Metabolomics reveals disrupted pathways in arginine synthesis and metabolism among ESCC patients

**FIGURE 3:**
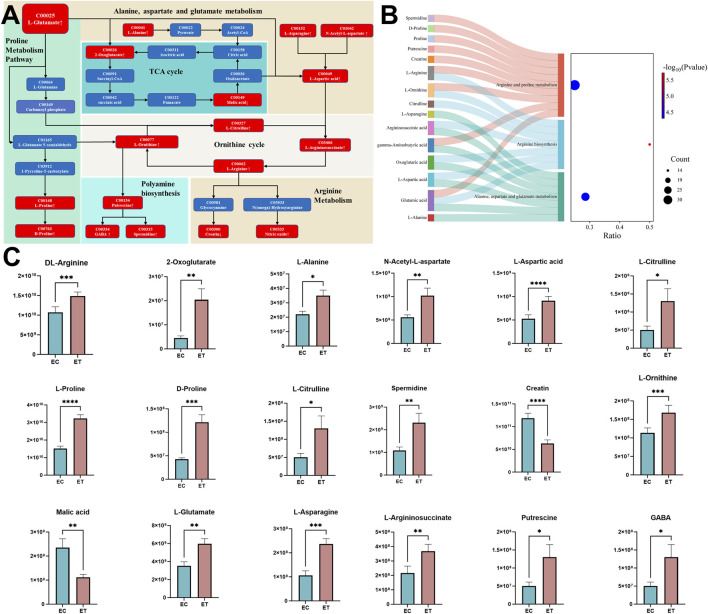
Arginine-related pathways are significantly enriched. **(A)** The crosstalk between amino acids metabolism (red boxes indicate DEMs; blue boxes indicate non-DEMs; upward arrows indicate metabolites upregulated in the ESCC group; downward arrows indicate metabolites downregulated in the ESCC group); **(B)** Sankey diagram; **(C)** Relative content of amino acids. *P < 0.05, **P < 0.01, ***P < 0.001, ****P < 0.0001.

Notably, the various arginine-related pathways, including arginine and proline metabolism, alanine, aspartate and glutamate metabolism, and arginine biosynthesis, were significantly enriched (Figure 2G), especially arginine biosynthesis. As shown in Figure 3, tumor tissues from ESCC patients exhibited significant dysregulation of arginine metabolism (Figures 3A,B). Key metabolites involved in the ornithine cycle, proline metabolism, the tricarboxylic acid (TCA) cycle, and polyamine biosynthesis were coordinately altered, with most showing significant upregulation (Figure 3C). It indicates that arginine metabolic reprogramming may be a key characteristic of metabolic dysregulation in ESCC.

In the arginine-proline metabolic axis, L-ornithine, L-arginine, L-citrulline, and arginosuccinate were markedly elevated, indicating enhanced flux through the arginine biosynthetic pathway. Concurrently, L-proline and D-proline were increased, reflecting a shunt of L-glutamate toward proline synthesis via 1-pyrroline-5-carboxylate. The alanine, aspartate, and glutamate metabolism pathway was also significantly enriched, with L-glutamate, L-aspartate, L-asparagine, and N-acetyl-L-aspartate showing increased abundance. These changes were linked to the TCA cycle, where two-oxoglutarate and malate were upregulated, suggesting a metabolic coupling between amino acid catabolism and energy production (Figure 3). As a key node in arginine metabolism crosstalk, DL-arginine significantly increased in ESCC tissues (Figures 3A,C). In addition, polyamine biosynthesis was activated, as evidenced by elevated levels of putrescine, spermidine, and γ-aminobutyric acid (GABA), all derived from L-ornithine. These findings reveal a profound reorganization of nitrogen and energy metabolism in ESCC, characterized by increased turnover of arginine and proline, accelerated polyamine synthesis, and altered citric acid cycle activity—which may promote tumor cell proliferation, survival, and stress adaptation.

Collectively, ESCC patients exhibit significant reprogramming of arginine metabolism, suggesting that dysregulation of the arginine–NO pathway is closely associated with ESCC progression.

### 3.4 HIF-1α-mediated NO metabolism is closely related to the development of ESCC

**FIGURE 4:**
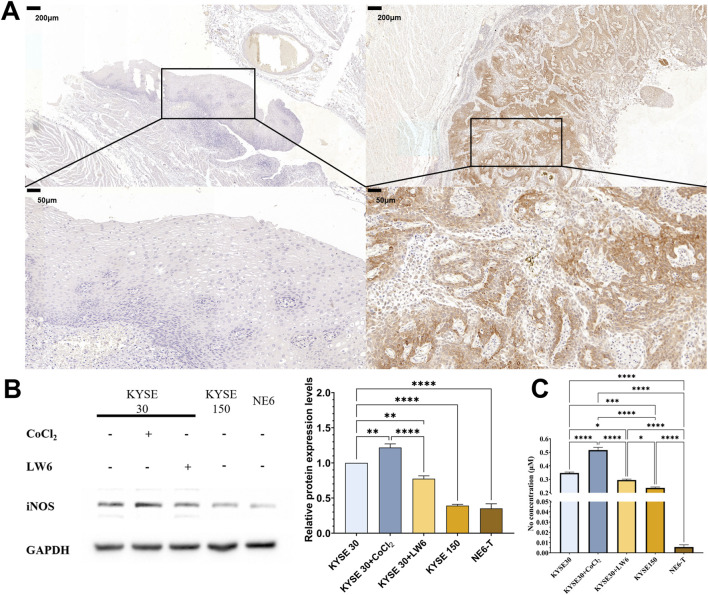
Quantitative detection of iNOS and NO. **(A)** Immunohistochemical staining results for iNOS protein in esophageal cancer tissue (right) versus normal tissue (left); fields of view at different magnifications are shown (scale bars: 200 μm, 50 μm); **(B)** WB analysis of iNOS protein expression in each experimental group, along with quantitative statistical results of corresponding band intensity values; **(C)** NO concentration measurement results. *P < 0.05, **P < 0.01, ***P < 0.001, ****P < 0.0001.

Given that arginine serves as a substrate for NO production through NOS-mediated catalysis, we performed IHC and WB analyses to preliminarily evaluate iNOS expression in ESCC at both the tissue and cellular levels. Immunohistochemical staining of 11 pairs of ESCC and matched adjacent non-cancerous tissue samples revealed that iNOS was primarily localized in the cytoplasm of cancer cells, with almost no significant positive expression observed in adjacent non-cancerous tissue (Figure 4A).

WB analysis revealed significantly higher levels of iNOS protein expression in ESCC cell lines compared to the NE6-T cell line (P < 0.05). Further treatment of KYSE-30 cells with 150 μmol/L CoCl_2_ (HIF-1α inducer) or 10 μmol/L LW6 (HIF-1α inhibitor) resulted in significantly increased iNOS protein expression and NO content in the CoCl_2_ group, while the LW6 group showed significant decreases, suggesting a positive correlation between HIF-1α and iNOS/NO expression. Additionally, WB quantitative analysis revealed significantly higher iNOS protein grayscale values in highly differentiated ESCC cells compared to poorly differentiated cells, suggesting that iNOS expression levels may correlate with the differentiation status of ESCC (Figures 4B,C).

**FIGURE 5:**
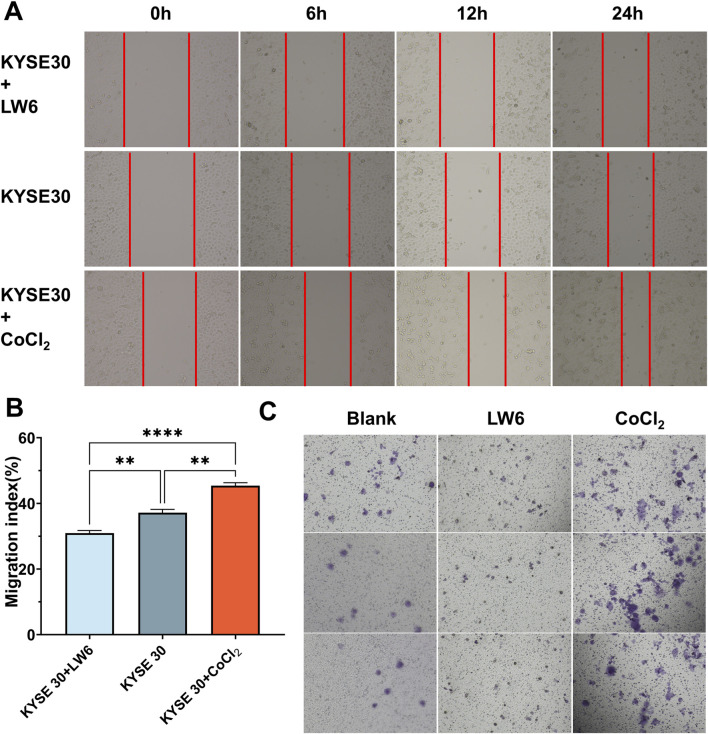
Invasion and Migration Assay. **(A)** Cell migration images of esophageal cancer cells in different treatment groups (red lines indicate initial scratch boundaries); **(B)** shows corresponding migration index quantitative bar charts for each group; **(C)** Invasion assay of EC cells in different treatment groups. *P < 0.05, **P < 0.01, ***P < 0.001, ****P < 0.0001.

Functional experiments were performed *in vitro* using the HIF-1α agonist CoCl_2_ and the HIF-1α inhibitor LW6. Wound healing and Transwell invasion assays were conducted to evaluate the role of the HIF-1α–iNOS–NO signaling pathway in regulating the migratory and invasive capacities of ESCC cells. As shown in Figure 5, the addition of the HIF-1α inducer CoCl_2_ significantly enhanced the invasion and migration capabilities of KYSE-30. Conversely, the addition of the HIF-1α inhibitor LW6 produced the inverse effect. Combined with WB and NO quantification data, the CoCl_2_ group demonstrated that CoCl_2_, acting as a HIF-1α inducer, increased iNOS protein and NO levels while simultaneously enhancing cell migration and invasion capabilities, exhibiting consistent trends. The LW6 group showed opposite trends. These findings indicate that HIF-1α promotes ESCC cell migration through the upregulation of iNOS expression, thereby supporting the regulatory role of the HIF-1α–iNOS pathway in the malignant progression of ESCC. Furthermore, HIF-1α-mediated NO metabolism may represent a promising therapeutic and diagnostic target for ESCC.

## 4 Discussion

This study employed untargeted metabolomics to profile metabolic alterations in ESCC tissues, identifying 2,850 metabolites with 939 differentially expressed metabolites (575 upregulated, 364 downregulated). Pathway enrichment analysis revealed 17 significantly altered metabolic pathways, among which arginine-related metabolism emerged as a predominant feature. By integrating molecular biology and functional assays, we demonstrated that the HIF-1α-iNOS-NO axis mediates this metabolic perturbation, thereby enhancing the migratory and invasive capacities of ESCC cells.

Arginine plays a key role in cancer progression. In hepatocellular carcinoma, tumor cells accumulate arginine by increasing its uptake and reducing its metabolic conversion, thereby driving uncontrolled proliferation and enhancing their ability to compete with immune cells for this critical nutrient (Mossmann et al., 2023). Furthermore, arginine induces tumor-associated macrophages to polarize toward a pro-tumor phenotype and suppresses the antitumor activity of CD8^+^ T cells, thereby reshaping the tumor microenvironment into a niche conducive to tumor growth (Zhu et al., 2025). These findings reveal that arginine not only supports tumor cell growth as a nutrient but also indirectly promotes tumor progression by reshaping the immune microenvironment. However, current research has yet to fully elucidate how arginine promotes malignant progression in EC.

Given that arginine serves as the substrate for NO production via nitric oxide synthases (NOS), we focused on the NOS-NO pathway. NO and NOS play crucial roles in esophageal diseases such as gastroesophageal reflux disease (McAdam et al., 2012), Barrett’s esophagus and EAC (Goldstein et al., 1998; Ferguson et al., 2008; Vaninetti et al., 2008), and ESCC (Tanaka et al., 1999; Kumagai et al., 2015). In humans, NOS is primarily categorized into nNOS, iNOS, and eNOS. A growing body of evidence suggests that NOS, particularly iNOS, is a key mediator in the pathogenesis of esophageal diseases. In benign esophageal conditions such as gastroesophageal reflux disease, exposure to gastric acid and bile acids induces iNOS expression. During malignant transformation and progression, iNOS becomes persistently overexpressed in both ESCC and esophageal adenocarcinoma. The resulting NO, together with its reactive nitrogen species derivatives, exerts mutagenic effects and disrupts normal cellular signaling pathways, thereby contributing to esophageal carcinogenesis (Tanaka et al., 1999; McAdam et al., 2012). Functional studies have shown that, in preclinical models, iNOS inhibitors reduce NO production and inhibit the formation of esophageal tumors (Chen et al., 2004). Clinically, patients with ESCC exhibit elevated NO levels and oxidative stress markers (Sayir et al., 2019), and studies have shown that NO promotes the development of Barrett’s esophagus by S-nitrosylation-dependent inhibition of Rho/ROCK signaling (Fujiya et al., 2022). Furthermore, serum metabolomics studies indicate that the L-arginine/NO pathway drives metabolic reprogramming in ESCC, with increased arginine bioavailability and overexpression of NOS being positively correlated with disease progression (Bednarz-Misa et al., 2020). Beyond the esophagus, NO has also been detected in the exhaled breath of breast cancer patients and serves as a predictive marker for radiation pneumonitis in patients with thoracic cancers (McCurdy et al., 2011; Segrado et al., 2025).

Despite these advances, the integrated role of the HIF-1α-iNOS-NO axis in orchestrating arginine metabolic reprogramming specifically in ESCC, and its consequent functional impact on migration and invasion, has not been systematically demonstrated through combined metabolomic and functional approaches. Given that advanced tumors typically exhibit high HIF-1α expression due to tissue hypoxia, and enhanced iNOS expression correlates with both the occurrence (Chen et al., 2012) and progression (Shi et al., 2016) of ESCC, we hypothesized that HIF-1α-driven iNOS upregulation promotes arginine metabolism toward NO production, thereby sustaining the migratory capacity of ESCC cells under hypoxic conditions.

To confirm this hypothesis, we treated the well-differentiated ESCC cell line KYSE-30 with LW6 (a HIF-1α inhibitor) (Naik et al., 2015) and CoCl_2_ (a HIF-1α inducer) (Munoz-Sanchez and Chanez-Cardenas, 2019), and compared iNOS expression levels with those in the poorly differentiated KYSE-150 cells and the immortalized normal esophageal epithelial NE6-T cells. Our results show that ESCC cells exhibit higher levels of iNOS under normoxic conditions compared to normal epithelial cells, and that expression levels are positively correlated with HIF-1α levels and the state of cellular differentiation. Interestingly, we observed that iNOS expression and NO production were higher in well-differentiated KYSE-30 cells compared to poorly differentiated KYSE-150 cells. This seemingly counterintuitive finding may reflect the retention of more intact metabolic regulatory machinery in well-differentiated cells, enabling a more robust NO response. Conversely, poorly differentiated cells may have undergone adaptive changes that alter NO dynamics. Current evidence suggests that NO exerts its pro-migratory and pro-invasive effects through multiple downstream signaling pathways. NO can S-nitrosylate cysteine residues on key signaling proteins (such as Src kinase and RhoA GTPase), altering their conformation and enzymatic activity (Ding et al., 2021). This process subsequently drives cytoskeletal reorganization, focal adhesion turnover, and pseudopod formation, thereby promoting directed cell migration (Srivastava and Premi, 2025). NO has been shown to downregulate E-cadherin expression while upregulating epithelial-mesenchymal transition (EMT) transcription factors such as vimentin, Snail, and Twist (Monteiro et al., 2019), promoting the transition of cells from an epithelial phenotype to a mesenchymal phenotype, thereby conferring enhanced invasive capacity (Kumari, 2026). NO can upregulate the expression and activity of MMP-2 and MMP-9. These enzymes degrade extracellular matrix (ECM) components (such as collagen IV), disrupt tissue barriers, and create pathways for tumor cell invasion (Clemons et al., 2010; Kumari, 2026). In this study, we observed that treatment with the HIF-1α inducer CoCl_2_ led to elevated iNOS/NO levels accompanied by enhanced migration and invasion capabilities, whereas the HIF-1α inhibitor LW6 produced the opposite effect, which is highly consistent with the mechanisms described above.

In summary, this study provides evidence that hypoxic ESCC cells can reprogram arginine metabolism via the HIF-1α-iNOS-NO axis, which can enhance ESCC cell migration and invasion capabilities. This effect is more pronounced in highly differentiated ESCC cells. Our findings suggest that NO may serve as a functional indicator of the metabolic state in ESCC; however, its clinical value as a diagnostic or prognostic biomarker requires further validation in larger cohorts and with available biological samples. Notably, the clinical translation of NO remains highly challenging due to its extremely short half-life of only a few seconds. Under physiological conditions, NO rapidly reacts with oxygen, superoxide, hemoglobin, and other substances to form stable derivatives such as nitrite and nitrate, making the direct detection of native NO molecules extremely difficult (Andrabi et al., 2023). Accurate NO detection remains challenging due to its extremely short half-life, low endogenous abundance, and interference from reactive nitrogen and oxygen species. Moreover, most conventional methods detect stable NO metabolites rather than NO itself, limiting real-time monitoring of NO dynamics and hindering mechanistic and clinical investigations.

The results of this study lay out multiple avenues for future research. First, the observed reprogramming of arginine metabolism needs to be validated in larger population cohorts to assess the potential of NO as a non-invasive biomarker. Second, the findings regarding the differential upregulation of iNOS in well-differentiated versus poorly differentiated ESCC cells, and their functional implications, warrant further investigation. Third, the extremely short half-life of NO limits its real-time detection; therefore, our team is developing electrochemical sensors for NO to dynamically monitor its release in biological models (Liu H. et al., 2025).

## 5 Conclusion

Untargeted metabolomics data indicate that significant amino acid metabolic reprogramming occurs in ESCC. Among these, arginine-related metabolic pathways exhibit notable metabolic dysregulation. This metabolic dysfunction stems from the abnormal activation of the HIF-1α-iNOS-NO axis in ESCC. By regulating HIF-1α, it is possible to suppress the enhanced cellular invasion and migration behaviors induced by arginine-NO metabolism. Furthermore, we have confirmed that NO is a potential tumor marker.

## Data Availability

The data presented in the study are deposited in the OMIX databasey, accession number OMIX018284, available at: https://ngdc.cncb.ac.cn/omix/release/OMIX018284.
